# Reply to Letter to the Editor

**DOI:** 10.1590/2175-8239-JBN-2022-0024-REP-en

**Published:** 2022-06-27

**Authors:** Ricardo Portiolli Franco

**Affiliations:** 1 Fundação Pró-Renal Brasil, Centro de Nefrologia Intervencionista, Curitiba, PR, Brasil.

Recently, we published an update regarding the concepts of the Fistula First Initiative and the latest evidence and guidelines on the indications of arteriovenous grafts (AVG) and fistulas (AVF) for hemodialysis (HD) vascular access^1^. The article was commented on in a letter to the editor by De Castro-Santos, who discussed the indication of AVG in patients with anticipated short duration on HD. De Castro-Santos addressed two points. First, he noted a difference in content between our manuscript and the KDOQI 2019 guidelines^2^ and stated: “In the article of the Brazilian Journal of Nephrology, the author considers indication of graft for patients with the probability of hemodialysis for less than one year. In the original document, the recommendation is for patients with survival time less than one year.” Second, he argues that Table 2 in our article is suggesting AVG for patients with an HD expectancy of more than one year.

We sincerely thank De Castro-Santos for his careful and critical reading of our paper, as it led us to find a diagraming error in the Portuguese version of the article. In this version, the signs for greater than (>) and less than (<) were reversed in the head row, causing the table columns to go against the KDOQI guidelines^2^. The second point is thus explained by this error and I apologize for not noticing this during the revisions.

The English version is correct, and Table 2 of the article states that for patients with HD expectancy of less than one year and a non-urgent start of HD, a forearm AVG or a brachiocephalic AVF (with a high likelihood of unassisted maturation) could be considered as the first choice. The table is an attempt to summarize Figures 1.2 and 1.3 and statement 3.1 B of the Guidelines (page S45) and present them in a more visual fashion.

The literal transcription of statement 3.1 B of the guideline is:

“B) A patient’s ESKD Life-Plan includes an anticipated limited duration (e.g., <1 year) on HD:

- Forearm loop AVG or brachiocephalic AVF (with high likelihood of unassisted maturation)
- Upper arm AVG”.

Therefore, the choice between AVG and AVF lies with the surgeon, if both have the same chance of unassisted maturation.

We agree with the first point made by De Castro-Santos that there is an important conceptual difference between “survival time less than one year” and “HD expectancy less than one year”, but as shown above, the table reproduced the terms of the guideline statement. We also agree with De Castro-Santos that “survival time” is a more appropriate interpretation of the guideline than “HD expectancy”. In our article, we try to make this clear: “In patients with an **estimated survival of less than one year**, the latest guidelines consider AVG or AVF with a high chance of maturation (i.e., brachiocephalic) as the first choice.”

De Castro-Santos brings an important and frequent example to the discussion in his letter: elderly patients with multiple comorbidities and contraindication to transplantation, who will probably remain on HD for more than a year. If this patient is already on HD using a catheter, he or she would probably benefit from an AVG, as it is more likely to mature and can be punctured without intervention. However, if the case is a non-urgent pre-HD patient, with a possibility of a brachiocephalic AVF, this might be a better choice as it would give us time to treat a primary failure if it occurs. The concept brought by the KDOQI 2019 guidelines is to individualize the choice while avoiding the creation of accesses whith a high chance of primary failure, which leads to longer catheter time.

As De Castro-Santos argues, inadequate use of AVG can lead to a higher number of interventions to promote and maintain patency and to higher health-related costs. Therefore, the decision about using an AVG or AVF should also take into account the accessibility to angioplasty and endovascular procedures. The overuse of AVG in low-resource settings where patients do not have access to angioplasty and thrombectomy can potentially lead to a high incidence of early intractable AVG thrombosis, due to the high frequency of venous anastomosis stenoses. Thus, the guidelines should be read in light of different realities and care infra-structure.

Once again, I would like to thank De Castro-Santos for pointing out the questionable terminology and for identifying the diagraming error in the Portuguese version, which will be formally corrected in an errata.
